# Prevalence of Depression in School-Going Adolescents and Its Impact on Scholastic Performance: A Cross-Sectional Study

**DOI:** 10.7759/cureus.38340

**Published:** 2023-04-30

**Authors:** Veni Nirudya, Mohan Reddy M, Ruth Sneha Chandrasekhar, Purushotham A, Mano Ranjitha V

**Affiliations:** 1 Psychiatry, Sri Devaraj Urs Academy of Higher Education and Research, Kolar, IND

**Keywords:** scholastic performance, phq-a, depression, ces-dc, adolescence

## Abstract

Background: Adolescents are more prone to depression, which needs early identification.

Aim: The aim of the study is to assess the prevalence of depression in school-going adolescents and its association with their scholastic performance.

Materials and methods: This questionnaire-based cross-sectional study was conducted on 473 students, among whom 271 girls and 202 boys with an age group of 12-17 years, who were selected through simple random sampling from four high schools located in and around Kolar city, Karnataka, India, from January 2023 to March 2023, were included in the study after obtaining three informed consents from adolescents. A questionnaire was given to all the study participants, and the depression was assessed according to the Centre for Epidemiological Studies Depression Scale for Children (CES-DC) score. The severity of depression among the children was assessed by the Patient Health Questionnaire-A (PHQ-A). Further, these children were assessed for their scholastic performance based on their final examination scores. The data were entered into an Excel sheet, and statistical analysis was performed.

Results: A total of 473 subjects were included in the present study; 177 had depression, which represents 37% of the total population. According to PHQ-A, 31% had mild depression, 5% had moderate depression, and 1% had severe depression. The association between depression and scholastic performance was not significant.

Conclusion: In the current study sample, depression was found to be mild to moderate, which reflects the overall performance of adolescents. All students were treated accordingly. Hence, active measures are to be initiated to prevent depression.

## Introduction

Depression, the most common mental illness among adolescents, is characterised by sadness, loss of interest, guilt, loss of appetite, and difficulty concentrating [1]. Globally, 34% of teenagers between the ages of 10 and 19 are at risk of developing clinical depression, which is greater than the estimates for those between the ages of 18 and 25 [2]. After cardiac and respiratory conditions, depression is the third-leading cause of disability. According to the Global Burden of Disease, one of the primary leading causes of years with a disability-adjusted life expectancy in 2020 will be depression [3].

For a transition from puberty to maturity, an individual must go through an intermediate stage of development known as adolescence [4]. Around the world, the prevalence of mental problems in children and adolescents ranges from 1 to 51%. India is home to 21% of the teenage population worldwide [5]. According to several community-based studies, 2.6% to 35.6% of kids suffer from psychiatric illnesses. According to WHO estimates, mental health disorders account for 16% of the worldwide illness burden and harm among adolescents aged 10 to 19 years [6,7]. Additionally, it states that depression frequently impacts family connections, social functioning, and academic achievement, and that most suicides in India are noticed in the under-30 age group [8]. Studies also report that depression often affects many functions in humans and is recurring in nature.

Hence, there is a need to study depression among school students, as the onset of most lifetime mental disorders occurs during this period. The study aimed to assess the prevalence of depression in adolescents and its association with scholastic performance.

## Materials and methods

Methodology

A total of 473 students, among whom 271 girls and 202 boys with an age group of 12-17 years, were chosen through simple random sampling from four high schools in and around Kolar City, Karnataka, India, and included in this questionnaire-based cross-sectional study from January 2023 to March 2023 after receiving three informed consents from participants, parents, and school administration.

All the students were administered a semi-structured questionnaire named Centre for Epidemiological Studies Depression Scale for Children (CES-DC) in English and explained in their mother tongue to assess depression. A psychiatrist evaluated the children who exhibit features suggestive of depression, and the diagnosis was confirmed.

The severity of depression among the children was assessed by the Patient Health Questionnaire-A (PHQ-A). Further, these children were evaluated based on their final exam scores for scholastic performance.

Statistical analysis

The data obtained were recorded in an Excel data sheet. All categorical data were presented using frequency and percentage, and all continuous measurements were summarized using mean ± SD. Each clinical and demographic factor was associated with clinical depression using the Chi-square test or Fisher's exact test based on the expected frequencies for categorical data and the independent sample T-test for a continuous variable based on the normality assumption. The P-value was considered significant at a 5% significance level for all analyses.

## Results

As shown in Table 1, a total of 473 students of age group 12-17 years were assessed for depression in the present study. The mean age of the students selected for the study was 14±1.22 years. Out of the total participants, male students accounted for 202 (42%), and female students accounted for 271 (57%). Most participants were from nuclear families, with a percentage of 69%. All the study participants were divided as per birth order and are tabulated in Table 1.

**Table 1:** Sociodemographic profile of the students

Variable	Number of study participants, n (%)
Sex	
Male	202 (42%)
Female	271 (57%)
Age of students (years)	
12	91 (19%)
13	114 (24%)
14	130 (27%)
15	91 (19%)
16	44 (9%)
17	11 (2%)
Family type	
Nuclear	330 (69%)
Joint	143 (30%)
Birth order	
First	214 (45%)
Second	211 (44%)
Third	46 (9%)
Fourth	2 (0.4%)

**Figure 1:**
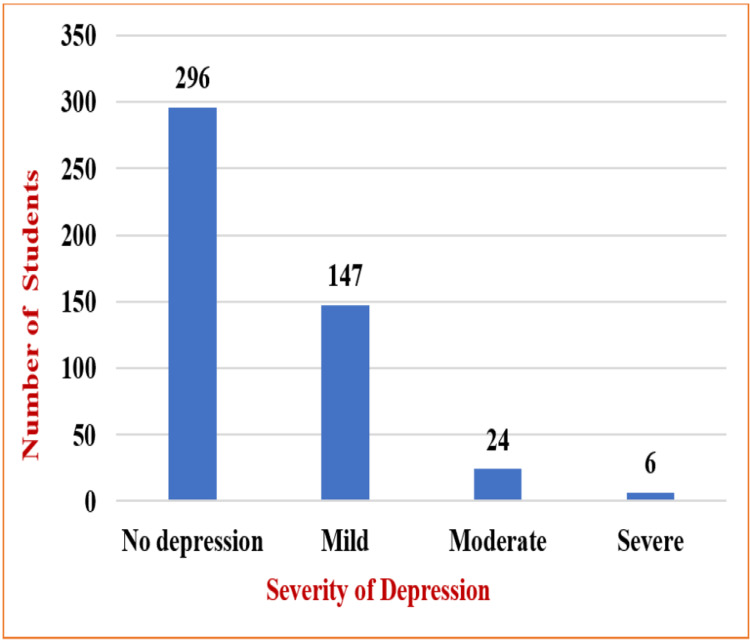
Prevalence of depression as per severity observed in PHQ-A results

As shown in Figure 1, the prevalence of depression according to the CES-DC was detected in 177 participants, representing 37% of the total population. According to the PHQ-A data, the prevalence of depression was as follows.

**Figure 2:**
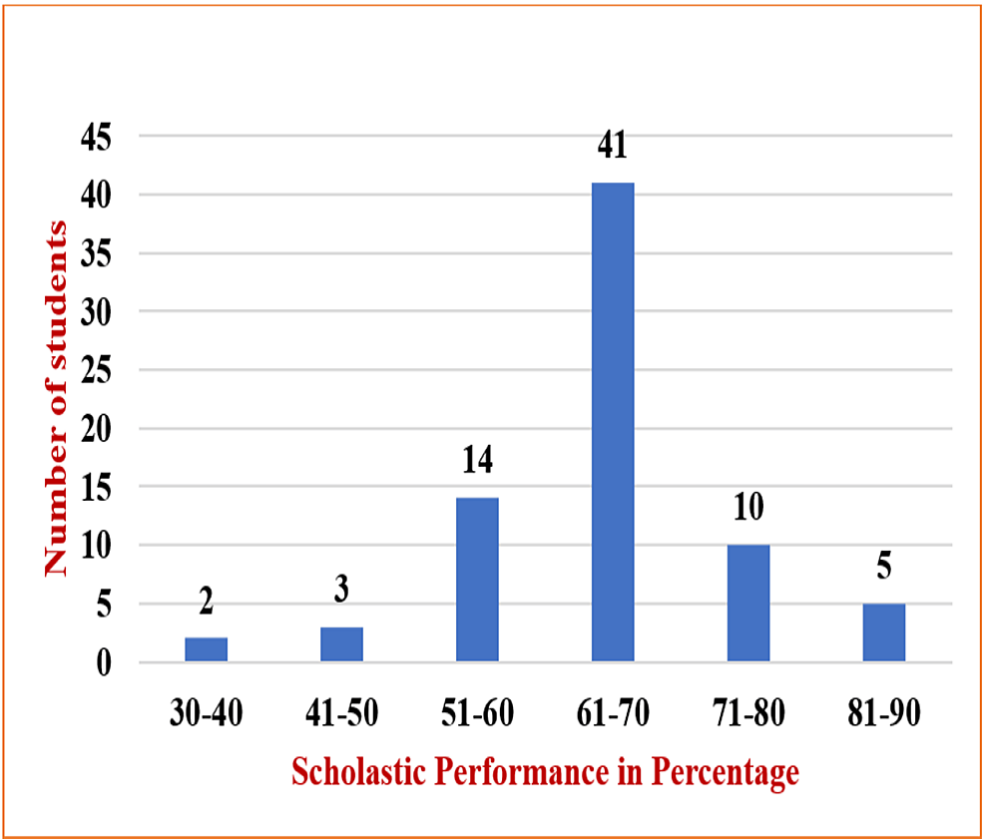
Scholastic performance of male students and depression

As depicted in Figure 2, the majority (n=41) of the male students with depression secured 61-70%, and only five secured above 80%.

**Figure 3:**
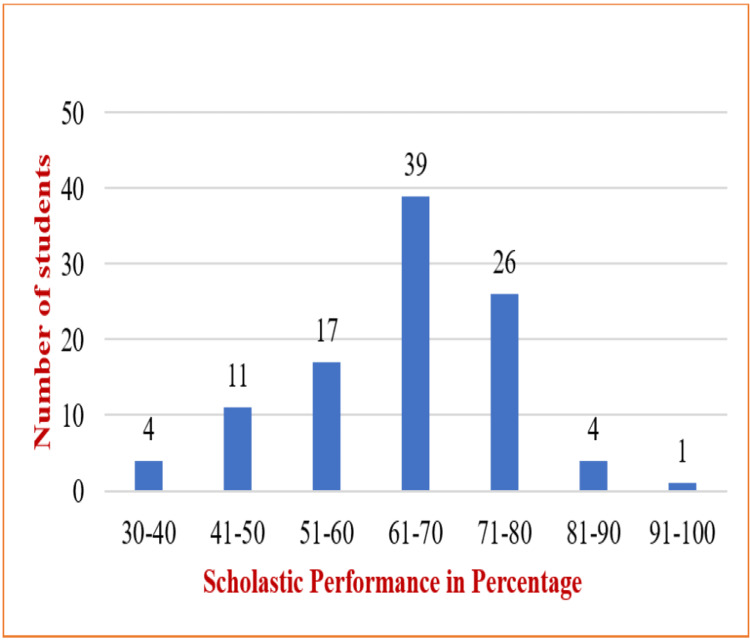
Scholastic performance of female students and depression

As depicted in Figure 3, the majority (n=39) of female students with depression secured 61-70%, four members secured above 81-90%, and one student secured above 91%.

According to Table 2, there was significant depression noted in overall students with p<0.05.

**Table 2:** Prevalence of depression in students with respect to (PHQ)-A results

Sex	No depression	Mild depression	Moderate depression	Severe depression	X^2^	df	P-value
Male	126	64	9	3	12.7	3	0.0001*
Female	173	83	15	3			

As shown in Table 3, there was no significant association between depression and scholastic performance with p>0.05.

**Table 3:** Association of depression and scholastic performance in students

Sex	Number of students having depression	Mean scholastic performance	Chi-square value	P-value
Male	75	63.8%	1.5	0.209
Female	102	64.9%		

**Figure 4:**
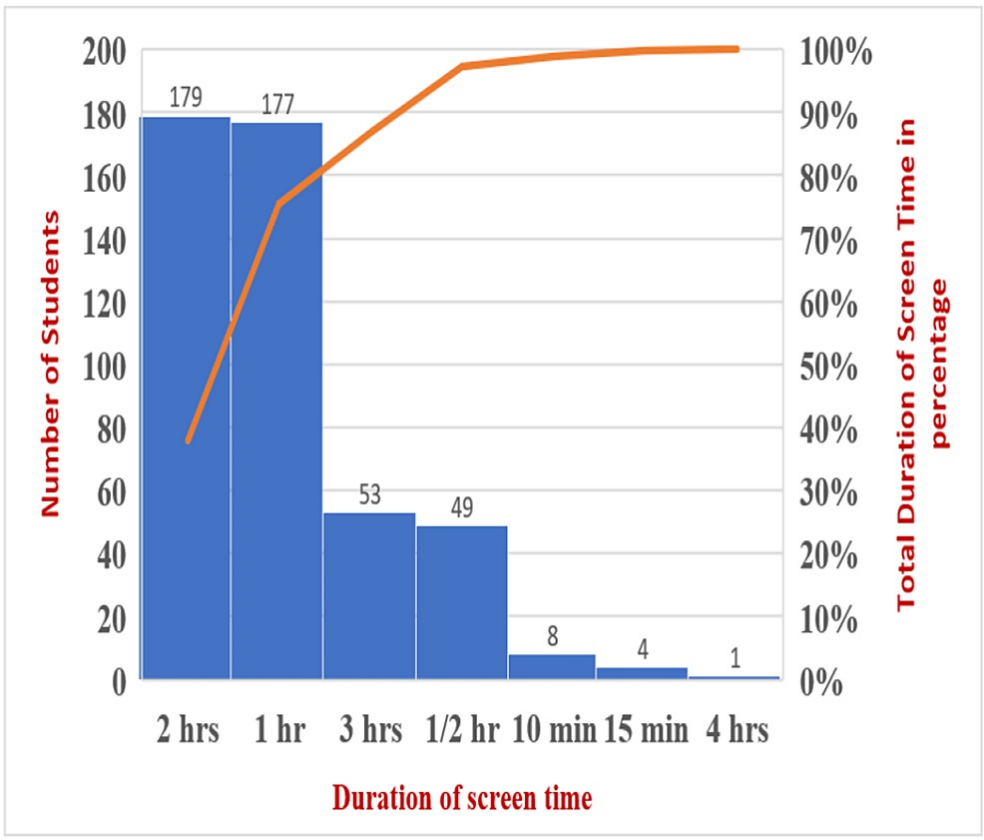
Number of participants spending screen time (Pareto chart showing descending order of frequencies with a cumulative line on secondary axis showing as a percentage of total)

As shown in Figure 4, among the total population, 37% had a screen time of two hours, 11% had a screen time of three hours, 10% had a screen time of half an hour, 1.6% had a screen time of 10 minutes, 0.8% had a screen time of 15 minutes, and 0.2% had a screen time of four hours, respectively.

As shown in Table 4, the Pearson correlation coefficient test was performed to find the correlation between individuals having depression and screen time. It was found that there was a negative correlation between individuals having depression and screen time spent, but the P-value was found to be significant P<0.00001.

**Table 4:** Correlation between screen time and depression

Number of individuals having depression	Screen time spent in hours	R-value	P-value
1	10 minutes	−0.6213	<0.00001*
2	15 minutes		
28	Half an hour		
72	1 hour		
53	2 hours		
13	3 hours		
1	4 hours		
2	5 hours		

## Discussion

An adolescent's social and personal lives may suffer as a result of the prevalence of depression. Because depression is a mental illness that affects young people, it is crucial to know how common it is among adolescents in school [9].

Depression was found to be mild in 31% of the study population, moderate in 5.4%, and severe in 1.2% of the high school students in the Kolar district.

Depression in school-aged teenagers was precipitated by broken households, including inter-parental confrontations, alcohol use by the father, solo parenting, and low self-esteem brought on by negative body views. The observed participants' overall depression rate was determined to be 37%, which is comparable to the 2017 Pokhara Metropolitan research [10].

The prevalence of depression was reported to be 50% in a few previous publications by Nagendra et al. and Jha et al., which is greater than the results of the current study [11,12]. The prevalence of depression among teenagers in other study reports in Indian literature ranged from 10% to 27%, which is lower than the results of the current study [13].

The common severity of depression in the present study was mild depression, noted in 31% of individuals, followed by moderate 5% and severe 1%, similar to study results conducted by Naushad et al. [14]. These results were contrary to the study results of Jha et al., who stated that moderate depression (41%) has the maximum number of participants, followed by mild (20%) and severe (11%).

Our study findings also showed a significant relationship between gender and the prevalence of depression, with depression slightly more common in girls than in boys (P<0.05). Despite the fact that both sexes reported using the internet or social media in harmful ways, females attributed their melancholy to having poor body image and low self-esteem. Our study findings were consistent with past study findings that depression was more prevalent in women than in men [15]. This was further demonstrated by Angold et al., who claimed that the hormonal changes associated with puberty are the reason why depression predominates more in women [16].

It was also found that depression was associated with birth order, as stated by Sidana et al. [17]. Few other reports have published that the prevalence of depression did not increase with age, which is correlated with the present study findings in Table 1 [13]. In our study, scholastic performance was not much affected, and this could be substantiated by the observation that many of the participants had features of mild depression that did not hinder their academic performance. The percentage of marks for boys and girls is represented in Figures 2-3. The results report that most participants with depression secured percentages of marks ranging from 60% to 70%.

Adolescents are exposed to the internet and social media due to changing technologies. According to our study, which examined students' screen time, 37% of them spent their time between one and two hours engaging themselves in screen activity, 11% for three hours, 10% for half an hour, 1.6% for 10 minutes, 0.8% for 15 minutes, and 0.2% for four hours, respectively. Teenagers who engaged themselves more in screen activity expressed their actions of loneliness, fewer interactions with family members, boredom, and being a single child. The easy accessibility of technology was also one of the confounding factors. Future research should focus on the relationship between problematic social media use and depressive symptoms in adolescents, which is a relatively new area of study. The Pearson correlation coefficient test was used to determine the relationship between depressed students and screen time. There was a negative correlation found between individuals with depression and screen time spent, but the P-value was found to be significant at P<0.00001, which is consistent with the Maras et al. 2015 study in which screen time was significantly associated with depressive symptoms [18].

In the study population of 473 students, 177 were found to be depressed, accounting for 37% of the total population. Depression was noted to be prevalent among high school students. However, no significant link was found between depression and academic performance. It could be explained better by the observation that the majority of them experienced mild depression and were able to function normally. Furthermore, children with severe depression [1.2%] had a significantly negative impact on their academic performance.

As a result, with an increasing number of people suffering from depression, more research is needed to find the ideal link between depression and academic achievement. It is also essential to create awareness among students, parents, and teachers about factors associated with depression.

Limitation

This study did not include a long-term follow-up with students. Other areas of functioning, like non-academic activities, social interaction, and relationships with friends and family, were not explored.

## Conclusions

The present study draws the conclusion that the overall prevalence of depression among school-going adolescents in the Kolar region is mild to moderate. There was no significant association between depression and scholastic performance.

Those students with depression were reassured and counselled, and a few who required pharmacotherapy were offered it accordingly. Further, it has been suggested to have frequent mental health camps in schools periodically to screen for mental disorders in school children, especially depression. This helps to identify a child with depression at an early stage and initiate remedial measures that would help the child improve their overall well-being and scholastic performance.

## References

[1] Mkize LP, Nonkelela NF, Mkize DL (1998). Prevalence of depression in a university population. Curationis.

[2] Shorey S, Ng ED, Wong CH (2022). Global prevalence of depression and elevated depressive symptoms among adolescents: A systematic review and meta-analysis. Br J Clin Psychol.

[3] Lopez AD, Murray CC (1998). The global burden of disease, 1990-2020. Nat Med.

[4] Rask K, Astedt-Kurki P, Laippala P (2002). Adolescent subjective well-being and realized values. J Adv Nurs.

[5] (2023). The global burden of disease: generating evidence, guiding policy. http://www.healthdata.org/policy-report/global-burden-disease-generating-evidenceguiding-policy-european-union-and-free.

[6] (2023). The World Health Report 2001: Mental health: new understanding, new hope. World Health Report.

[7] (2023). Depression. https://www.nimh.nih.gov/health/publications/depression.

[8] Williams CA (1989). Empathy and burnout in male and female helping professionals. Res Nurs Health.

[9] Bharati DR, Kumari S, Prasad N, Choudhary SK, Kumar S, Pal R (2022). Correlates of depression among school going adolescents in the urban area of Patna in eastern India. J Family Med Prim Care.

[10] Poudel A, Lamichhane A, Magar KR, Khanal GP (2022). Non suicidal self injury and suicidal behavior among adolescents: co-occurrence and associated risk factors. BMC Psychiatry.

[11] Nagendra K, Sanjay D, Gouli C, Kalappanavar NK, VinodKumar CS (2012). Prevalence and association of depression and suicidal tendency among adolescent students. Int J Biomed Adv Res.

[12] Jha KK, Singh SK, Nirala SK, Kumar C, Kumar P, Aggrawal N (2017). Prevalence of depression among school-going adolescents in an urban area of Bihar, India. Indian J Psychol Med.

[13] Ekundayo OJ, Dodson-Stallworth J, Roofe M, Aban IB, Kempf MC, Ehiri JE, Jolly PE (2007). Prevalence and correlates of depressive symptoms among high school students in Hanover, Jamaica. ScientificWorldJournal.

[14] Naushad S, Farooqui W, Sharma S, Rani M, Singh R, Verma S (2014). Study of proportion and determinants of depression among college students in Mangalore city. Niger Med J.

[15] Asal AR, Abdel-Fattah MM (2007). Prevalence, symptomatology, and risk factors for depression among high school students in Saudi Arabia. Neurosciences (Riyadh).

[16] Angold A, Costello EJ, Erkanli A, Worthman CM (1999). Pubertal changes in hormone levels and depression in girls. Psychol Med.

[17] Sidana S, Kishore J, Ghosh V, Gulati D, Jiloha R, Anand T (2012). Prevalence of depression in students of a medical college in New Delhi: a cross-sectional study. Australas Med J.

[18] Maras D, Flament MF, Murray M, Buchholz A, Henderson KA, Obeid N, Goldfield GS (2015). Screen time is associated with depression and anxiety in Canadian youth. Prev Med.

